# Comparative study of mesenchymal stem cells from rat bone marrow and adipose tissue

**DOI:** 10.3906/biy-1802-52

**Published:** 2018-12-10

**Authors:** Qing HE, Zhaoyang YE, Yan ZHOU, Wen-Song TAN

**Affiliations:** 1 State Key Laboratory of Bioreactor Engineering, East China University of Science and Technology , Shanghai , P.R. China

**Keywords:** Adipose tissue, bone marrow, comparative analysis, long-term culture, mesenchymal stem cells

## Abstract

Several therapeutic products based on mesenchymal stem cells (MSCs) have been translated into clinical applications. MSCs should undergo in vitro culture before a sufficient quantity can be achieved. Hence, both expansion kinetics and the biological characteristics of derived cells from primary culture are pertinent to their applications. In the present study, MSCs were isolated from rat bone marrow and adipose tissue (designated as bMSCs and aMSCs, respectively) and cells were comparatively analyzed regarding cell morphology, proliferation, colony formation, differentiation potential, and immunophenotype following the long-term subculture. No apparent differences could be noticed concerning the morphology between bMSCs and aMSCs. The long-term subculture made both types of cells smaller, weakened their colony-forming ability, and stimulated the proliferation rate. However, bMSCs demonstrated better proliferation and colony-forming ability than aMSCs. No significant difference was observed about the expression of some immunophenotypes (i.e. CD29+/CD90+/CD34-/CD45-) regardless of cell types or population doublings. Notably, bMSCs, but not aMSCs, maintained the differentiation potential well after the long-term subculture. The present study demonstrates that MSCs derived from different tissues can be well expanded for the long term, although cells display gradually declined self-renewal and differentiation potentials to different extents depending on the tissue origins.

## 1. Introduction

Regenerative medicine is a growing field that aims to treat currently unmet clinical indications such as diabetes, cardiovascular disease, and neurological disorders by restoring or maintaining tissue function (Heathman et al., 2015) . Mesenchymal stem cells (MSCs) have tremendous potential for applications in regenerative medicine due to the abundant availability and potentials of self-renewal and differentiation (Lim et al., 2016) . Since Pittenger et al. demonstrated that human bone marrow-derived MSCs could be successfully induced to undergo multilineage differentiation in 1999 (Pittenger et al., 1999) , thousands of studies have been carried out with the objective of translating MSCs in clinical settings. Moreover, MSCs have been discovered to bear the capability of secreting a plethora of bioactive factors that are involved in immunomodulation, chemotaxis, apoptosis, antibfirosis, etc. (Mizukami et al., 2016) . It was also reported that MSCs that survived in vivo had the characteristics of pericytes and could maintained vasomotion, vascular maturation, and regulation of extracellular matrix turnover through mechanisms similar to the signal transduction between paracrine and other tissues (Dijk et al., 2015). According to the latest update at ClinicalTrials.gov, there were >800 trials registered related to MSCs as of 15 July 2018. To date, a few therapeutic MSCs products, including Prochymal (Osiris), ChondroyCelect (TiGenix), and MPC (Mesoblast), have been approved for clinical application in the United States, Europe, and Australia, respectively. In Korea, Hearticellgram (FCB-Pharmicell) and Caristem and Cuepistem (Osiris) were also made available in clinics. These products are used for the treatment of graft-versushost disease, cartilage injury, acute myocardial infarction, Crohn disease, etc.

Although the successful application of MSCs in clinical settings has become a reality, there are still many challenges associated with developing MSCs-based therapeutic products, especially concerning the production process of cell products. The MSCs culture specifics, including methods of isolation, expansion, cell enrichment, cell storage, and procedures for adherent or suspension cultures, play an important role in the production of MSCs-based products (dos Santos et al., 2013) . A strictly controlled process is the prerequisite for both safety and efficacy of MSCs-based products and the establishment of a standard protocol that meets the guidelines of good manufacturing procedures remains unresolved. In clinical treatment, cellbased therapy requires a high number of MSCs, typically more than one million cells per kilogram of the patient’s body weight. Given the extremely low frequency of MSCs of tissue origins (Fuchs et al., 2004) , an efficient ex vivo expansion process is required to attain such a large dose of cell products. In addition, in the case of developing one on the-shelf cell product for many individuals, an even larger quantity of cells is anticipated from one tissue sample. However, the challenge is that cells are very unique on their own, which places a hurdle for developing a general production process of all cell products. The expansion characteristics can be drastically varied related to the developmental stage of the cell’s tissue of origin, species, culture process, and so on (Deasy et al., 2005). Moreover, numerous studies have demonstrated that the growth rate, phenotype, and differentiation potential of MSCs can be altered after long-term culture, which definitely would exert great influences on the therapeutic effects of end products (Bara et al., 2014) . For instance, several studies demonstrated that the expression of senescence-associated β-galactosidase (SA β-gal, a senescence marker) in MSCs increased after long-term culture, which led to gradual senescence of MSCs (Li et al., 2012; Gu et al., 2016) . Others indicated that MSCs sustained prolonged self-renewal and homogeneous characteristics for over 50 passages without losing their differentiation potential (Meirelles and Nardi, 2003) . However, understanding of the effects of in vitro expansion on the characteristics of MSCs remains in its infancy, which is critical to cell-based therapeutic applications and warrants further thorough investigation.

An optimal source of MSCs for in vitro expansion is critical for developing cell-based therapeutics as MSCs can be derived from various tissues, such as bone marrow, umbilical cord blood, adipose tissue, etc. MSCs from different tissues display significant differences in characteristics and functions (Chen et al., 2015). For instance, bone morrow-derived MSCs (bMSCs), the earliest discovered and defined MSCs, showed a better proliferative capacity than other types of MSCs (dos Santos et al., 2014) . While in the past few decades, bMSCs have attracted great attention, the harvest of bone marrow is a highly invasive procedure. Umbilical cord blood is an alternative choice, which can be obtained by a less invasive method, without harm for the mother or the infant. However, whether full-term umbilical cord blood can serve as a source for isolating MSCs still remains controversial (Mareschi et al., 2001) , and MSCs derived from umbilical cord blood might be inefficient in undergoing adipogenesis (Kern et al., 2006) . Additionally, it is only limited to newborns. Adipose tissue represents a very attractive source of MSCs, due to its easy accessibility with a less invasive method (Kanth et al., 2015; Nishigaki et al., 2017) .

**Figure 1:**
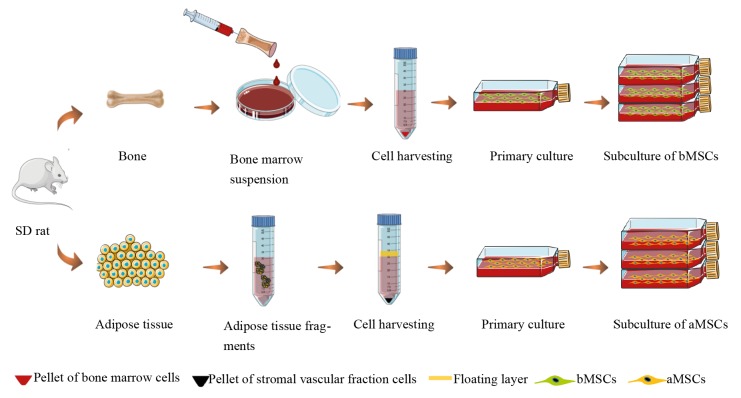
Schematic illustration of cell isolation and subculture processes.

Source-dependent and donor-dependent differences of MSCs properties, including implications on their clinical application, are still largely unknown. In the present study, our aim is to comparatively assess the properties of MSCs isolated from adipose tissue and bone marrow and the effects of long-term ex vivo expansion on these MSCs. As illustrated in Figure 1, rat bMSCs and adipose tissue derived MSCs (aMSCs) were isolated and cell morphology, proliferation, colony formation, differentiation capacity, and immune phenotype were examined for these cells during long-term culture.

## 2. Materials and methods

### 2.1. Isolation and primary culture of bMSCs and aMSCs

Bone marrow and adipose tissue were from 4-weekold male Sprague Dawley rats purchased from Shanghai Slac Laboratory Animal Co. All animal experimental procedures were performed in accordance with the guidelines for the care and use of laboratory animals at the Shanghai Laboratory Animals Center and the protocols were approved by the Institutional Animal Care and Use Committee of Shanghai Laboratory Animal Center.

Rat bMSCs were isolated as illustrated in Figure 1 as described by Huang et al. (2015) . Briefly, the rat’s hind limb femur and tibia were harvested, and after removal of the peripheral muscle tissue, both femur and tibia were soaked with alcohol briefly and then rinsed twice with phosphate-buffered saline (PBS) containing 1% penicillin/ streptomycin. Both ends of the epiphyseal were pinched of with a broken bone clamp and bone marrow was flushed out with α-minimum essential medium alpha medium (α-MEM, GIBCO) containing 10% fetal bovine serum (FBS, Biosun) until the flushing medium turned from brown to an orange-red color. The bone marrow suspension was filtered with a 200-mesh sieve and then centrifuged. The pellets were resuspended in growth medium composed of α-MEM supplemented with 10% FBS, and cells were plated in T25 flasks followed by incubating at 37 °C and 5% CO2. Initial medium refreshment was performed after 48 h and, after that, the medium was changed every 3 days until cells reached 80%–90% confluence.

Rat aMSCs were isolated as illustrated in Figure 1 as described previously (Sugii et al., 2011) . Rat bilateral inguinal adipose tissues were taken and soaked in alcohol briefly. After rinsing with PBS containing 1% penicillin/ streptomycin twice, adipose tissue was minced into small pieces and then treated with 2% collagenase II (Invitrogen) on a shaker at 120 rpm at 37 °C and 5% CO2 in an incubator. After 60 min, adipose tissue fragments turned into a thin cloud and mist and growth medium with 10% FBS was added to stop enzymatic digestion. The suspension was further pipetted 30–40 times and then filtered with 65-mesh and 150-mesh sieves in turn. The filtrate was centrifuged for 5 min at 1500 rpm and then the upper floating layer containing mature adipocytes and aqueous supernatant was carefully aspirated to obtain cell pellets, which were composed of stromal vascular fraction cells. The pellet was resuspended in 10 mL of PBS and cells were plated in T25 flasks in α-MEM with 10% FBS followed by incubating at 37 °C and 5% CO2. Initial medium refreshment was performed after 48 h and, after that, the medium was changed every 3 days until cells reached 80% to 90% confluence.

### 2.2. Long-term culture of bMSCs and aMSCs

Both bMSCs and aMSCs were subcultured until passage 3 and then used for subsequent long-term culture experiments. To initiate the long-term culture, bMSCs or aMSCs of passage 3 were plated at 5000 cells/cm2 in T25 flasks in growth medium. When cells reached 80% to 90% confluence, they were harvested via enzymatic treatment with 0.25% trypsin/EDTA (GIBCO), counted, and subcultured at 5000 cells/cm2 in T25 flasks. This process was repeated until 40 passages were achieved. During culture, the medium was refreshed every 3 days. For each passage, population doubling (PD), population doubling time (PDT), and cumulative population doublings (CPDs) were calculated based on cell counting and culture time based on the following equations, respectively:

$\mathrm{PD}=\log_{2}\frac{N_{n}}{N_{0}}$

$\mathrm{PDT}=\frac{T}{\mathrm{PD}}$

$\mathrm{CPD}=\overset{n=n}{\underset{n=4}{\Sigma }}{\mathrm{PD}}_{n}$

Here, n is passage number, N0 is cell number at seeding, Nn is cell number of the nth passage at harvesting, PDn is population doubling of the nth passage, and T is culture time in the respective passage.

In addition, at early passages, bMSCs or aMSCs plated in 24-well plates at 5000 cells/cm2 were also counted every other day and the growth curves were plotted for each passage of cells.

### 2.3. F-actin staining

Cell morphology was evaluated by F-actin staining. In 24well plates, bMSCs or aMSCs of passage 4 or 40 were plated at 5000 cells/cm2 and, after 24 h, cells were rinsed with PBS, fixed with 4% paraformaldehyde in PBS, and permeated with 0.1% Triton X-100 in PBS. After rinsing with PBS again, cells were treated with 2 μg/mL DAPI (Invitrogen) and 5 μg/mL rhodamine phalloidin (Invitrogen) for 30 min in the dark. Cells were observed and photographed under an inverted florescence microscope (Eclipse Ti-S, Nikon).

### 2.4. Colony formation assay

The frequency of colony-forming unit fibroblasts (CFUFs) for bMSCs or aMSCs was determined by colony formation assay. Cells of passage 4, 13, or 40 were plated in 6-well plates at 100 cells/well and the medium was refreshed twice a week. After 2 weeks, cells were rinsed with PBS, fixed with 4% paraformaldehyde in PBS, and stained with 0.1% crystal violet for 30 min. After rinsing with PBS again, the number of CFU-Fs was counted. All assays were performed in 6 replicates.

### 2.5. Flow cytometric analysis

Flow cytometry was applied to study the immunophenotype of cells. Approximately 1 × 10^6^ bMSCs or aMSCs at different passages were harvested and resuspended in growth medium to prepare single-cell suspensions. Cell preparations were incubated with 1 μg of the specific antibodies or corresponding isotype control antibodies as shown in the Table for 30 min at 4 °C in the dark. For CD73, the nonflourescence labeled primary antibody, cell preparations were subsequently treated with the secondary antibody, FITC rat antimouse IgG1 (BD), for 30 min at 4 °C. All cell preparations were analyzed by flow cytometry (FACSCalibur, BD) and data analysis was performed with FlowJo software (Tree Star).

**Table:** Antibody specifics for flow cytometric analysis.

Antigen	Primary antibody	Secondary antibody	Isotype control antibody	Source
CD29	FITC Hamster antirat CD29	--	FITC hamster IgM , λ1	BD
CD105	Alexa Fluor 405 mouse antirat/human CD105	--	Alexa Fluor 405 mouse IgG2A	Novus Biologicals
CD73	Purified mouse antirat CD73	FITC rat antimouse IgG1	FITC rat IgG1, κ	BD
CD44	FITC-conjugated mouse antirat CD44	--	FITC mouse IgG1	Abd Serotec
CD106	FITC-conjugated mouse antirat CD106	--	FITC mouse IgG1	Abd Serotec
CD90	FITC-conjugated mouse antirat CD90	--	FITC mouse IgG1, κ	BD
CD45	FITC-conjugated mouse antirat CD45	--	FITC mouse IgG1, κ	BD
CD34	Alexa Fluor 488 rabbit antirat CD34	--	Alexa Fluor 488 rabbit IgG1	Abcam

### 2.6. Induction of linage-specific differentiation

Evaluation of the differentiation potential of MSCs towards osteogenesis and adipogenesis was performed as described previously. Briefly, bMSCs or aMSCs were plated in 24-well plates at 1000 cells/cm2 and maintained in growth medium until 80% to 90% confluence. For osteogenic induction, cells were exposed to osteogenic induction medium composed of DMEM-HG supplemented with 10% FBS, 10 mM sodium β-glycerophosphate, 50 mg/L L-diphosphate ascorbic acid, 0.1 μmol/L dexamethasone, and 1% penicillin/streptomycin, and for adipogenic induction, cells were incubated in adipogenic induction medium of DMEM-HG supplemented with 10% FBS, 10 mg/L insulin, 0.1 mmol/L IBMX, 0.1 mmol/L indomethacin, 1 μmol/L dexamethasone, and 1% penicillin/streptomycin. After induction for 7, 14, or 21 days, cells were fixed with 4% paraformaldehyde in PBS. To assess osteogenic differentiation, cells were stained with Alizarin Red S (Sigma-Aldrich), and to assess adipogenic differentiation, lipid vacuoles in differentiated cells were stained using Red Oil O (Sigma-Aldrich).

### 2.7. Statistical analysis

Quantitative data were reported as averages ± standard deviations, unless otherwise stated. Statistical analyses were performed using Student’s t-test (GraphPad Software) and P < 0.05 was considered statistically significant.

## 3. Results

### 3.1. Morphological characterization of cells in primary and long-term cultures

**Figure 2:**
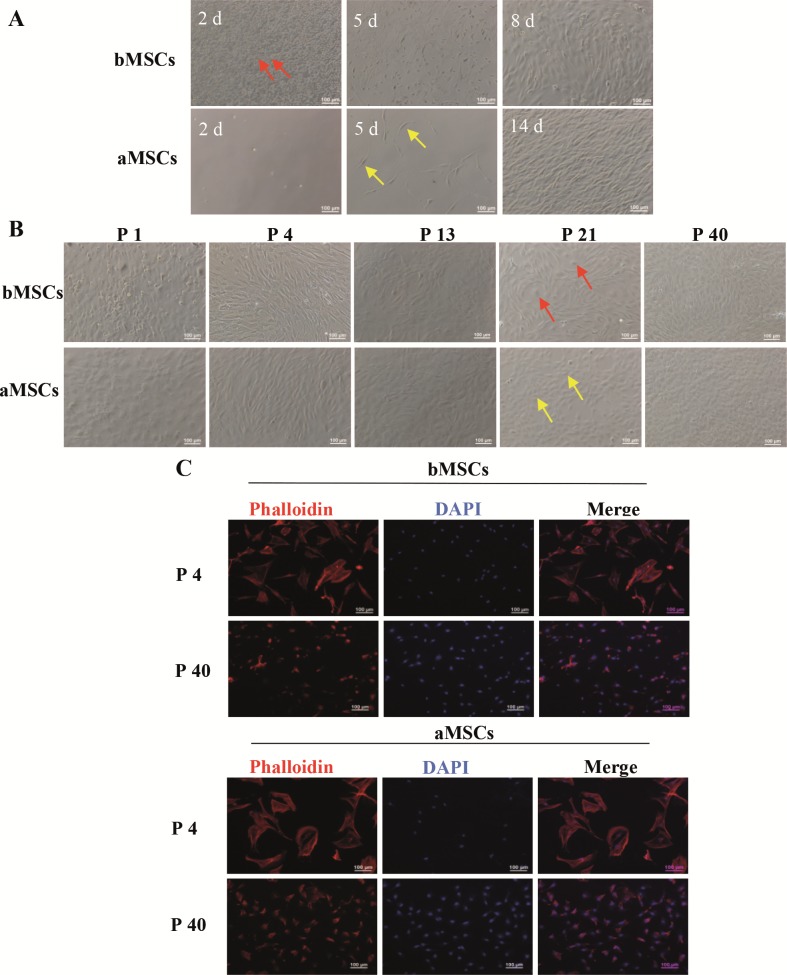
The morphology of bMSCs and aMSCs in primary and long-term cultures. A) Phase-contrast images of primary culture after different time intervals. The cells pointed out by the red arrow and the yellow arrow are spindle-shaped bMSCs and aMSCs, respectively. B) phase-contrast images after 6 days in culture at different passages. The cells pointed out by the red arrow and the yellow arrow are rounded cells and oval cells, respectively. C) Fluorescence images of F-actin staining after 24 h in culture at passages 4 and 40.

Primary cell culture was initiated after the bone marrow suspension was centrifuged and adipose tissue was enzymatically digested to release cells. During the culturing process, cells were observed and photographed under a phase-contrast microscope. In terms of cells from bone marrow, most cells were round on day 2, and only a few cells appeared elongated in shape (Figure 2A, red arrow). With prolonged culture time, the spindleshaped cells reached 50%–60% confluence on day 5 and ~90% confluence on day 8. For cells derived from adipose tissue, far fewer cells were present on day 2 and some spindle-shaped cells appeared on day 5 (Figure 2A, yellow arrow). On day 14, the spindle-shaped cells reached ~90% confluence. Therefore, for rats, bone marrow contained many more adhered cells in primary culture than inguinal adipose tissue.

Following subculture once (i.e. passage 1), both cultures contained cells with various shapes, such as round, polygonal, and elongated cells. After further subculture, at passage 4, cells displayed a uniform fibroblast-like morphology and tended to follow an ordered, aligned arrangement. At passage 13, bMSCs and aMSCs became shorter and wider and sparse nodules of clustered cells showed up in culture. At passage 21, some polygonal cells appeared in bMSCs (Figure 2B, red arrow) and cobblelike cells in aMSCs (Figure 2B, yellow arrow). Almost all bMSCs and aMSCs turned cobble-like at passage 40 and bMSCs were in general elongated more than aMSCs. These data suggested that during the long-term culture, the morphology of both bMSCs and aMSCs evolved following subculture.

In order to observe cell morphology more clearly, the cytoskeleton of bMSCs and aMSCs at passages 4 and 40 was further analyzed by F-actin staining. As shown in Figure 2C, at 24 h, bMSCs and aMSCs were spread out and uniformly distributed. DAPI-stained cell nuclei showing blue flourescence located in the center of cells and myofilament proteins as indicated in red fluorescence. Both bMSCs and aMSCs of passage 4 were large and showed a triangular or polygonal morphology. However, both bMSCs and aMSCs at passage 40 become smaller and displayed small, round, spindle, or polygonal morphologies. No apparent difference in morphology between bMSCs and aMSCs was noted.

### 3.2. Long-term subculture of bMSCs and aMSCs

**Figure 3:**
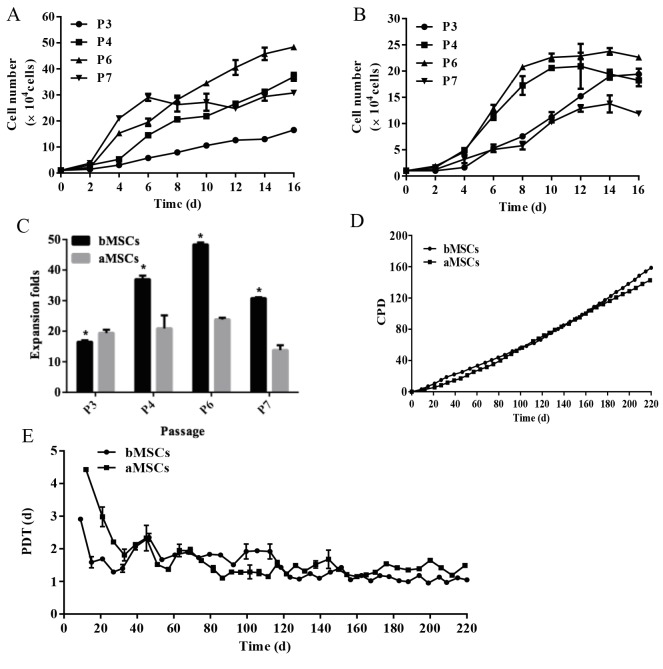
Growth characteristics of bMSCs and aMSCs during subculture. A) The growth curves of bMSCs at early passages. B) The growth curves of aMSCs at early passages. C) The expansion folds of bMSCs and aMSCs at early passages. D) CPD during long-term subculture. E) PDT during long-term subculture. *: P < 0.05 compared to aMSCs (n = 3).

At the early passages (before passage 7), the growth kinetics of bMSCs and aMSCs were as shown in Figures 3A and 3B, respectively. In general, both cell types demonstrated great proliferative potential at all different passages, showing a drastic increase in cell number with culture time within each passage. However, the growth profiles varied among different passages for both cell types. For bMSCs, within the first 6 days in culture at all examined passages, the growth rates increased with the increase of passage number, and after day 6, cells at passage 7 reached a stable plateau while cells at other passages were still in a proliferative state until day 16. For aMSCs, cells at passages 3 and 7 shared a similar profile with a slow proliferation rate, reaching the plateau after 14 days in culture. In contrast, aMSCs at passages 4 and 6 displayed much faster growth within the first 8 days in culture and then reached the plateau. As shown in Figure 3C, after 16 days of culture, bMSCs at passages 3, 4, 6, and 7 had expanded 16.5 ± 0.5, 37.0 ± 1.2, 48.4 ± 0.6, and 30.8 ± 0.3 times, respectively, while aMSCs had expanded 19.4 ± 1.1, 20.9 ± 4.3, 23.8 ± 0.6, and 13.8 ± 1.6 times, respectively. These data suggested that within passage 7, the initial subculture promoted cell proliferation and cell proliferative potential declined with extended subculture for early passages of cells. Moreover, bMSCs had higher proliferative potential than aMSCs.

Furthermore, the proliferation characteristics (i.e. PD, CPD, and PDT) of MSCs were analyzed during the longterm subculture for a total of 220 days. Overall, bMSCs and aMSCs underwent a significant expansion of 158 and 142 PD, respectively (Figure 3D), suggesting great proliferative potential for both cell types. In other words, 2.8 × 10^51^ cells and 4.1 × 10^46^ cells could be obtained from 5000 cells of bMSCs and aMSCs at passage 3, respectively. Thus, a much greater quantity of MSCs could be acquired from the same starting seed cell number for bMSCs than aMSCs. PDT was further calculated to analyze the growth rate of cells at each passage. In general, the PDT of bMSCs and aMSCs decreased drastically from 3.0–4.5 days to 1.5–2.5 days within 30 days, followed by a steady decrease to 1.0–2.0 days until the end of the long-term subculture (Figure 3E). However, at the early stage (<40 days), the PDT of aMSCs was greater than that of bMSCs, indicating that during this period, bMSCs proliferated better than aMSCs. Following extended subculture, the PDT of bMSCs became slightly higher than that of aMSCs between 70 and 120 days and this trend was then reversed after 120 days of subculture. Hence, it was demonstrated that both bMSCs and aMSCs had great proliferative potential during long-term subculture and tended to expand faster with subculturing.

### 3.3. Colony-forming capability of bMSCs and aMSCs after subculture

**Figure 4:**
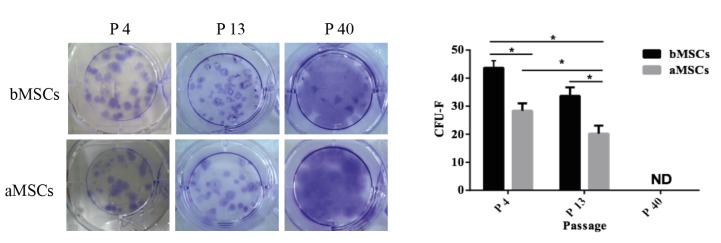
CFU-Fs of bMSCs and aMSCs. At different passages, bMSCs and aMSCs were plated in 6-well plates at 100 cells/well for 2 weeks and then stained with crystal violet. A) Images of crystal violet staining; B) quantification of CFU-Fs. ND: Not detected; *: P < 0.05 (n = 6).

As defined, MSCs should be able to form single cell derived colonies when plated at a low density (Colter et al., 2000). The CFU-Fs assay is often applied to determine the self-renewal potential of stem cells. At different passages, CFU-Fs of bMSCs and aMSCs were recorded and compared. As illustrated in Figure 4A, after 14 days of culture at a low density of 10 cells/cm2, positive crystal violet staining was detected for both bMSCs and aMSCs at passages 4 and 13, suggesting that bMSCs and aMSCs had self-renewal potential. Notably, as shown in Figure 4B, at both passages 4 and 13, the numbers of CFU-Fs for bMSCs were significantly higher than those of aMSCs (43.7 ± 2.4 vs. 36.7 ± 2.8 at passage 4; 28 ± 2.4 vs. 20.2 ± 2.6 at passage 13). Furthermore, compared to those at passage 13, both types of MSCs at passage 4 had a statistically higher number of CFU-Fs. Interestingly, for bMSCs and aMSCs at passage 40, the crystal staining diffused all over the plate, possibly due to rapid cell proliferation, such that it became impractical to count CFU-Fs accurately. These data demonstrated that both bMSCs and aMSCs preserved the self-renewal potential following subculture and bMSCs were better than aMSCs at self-renewal.

### 3.4. Lineage-specific differentiation of bMSCs and aMSCs

**Figure 5:**
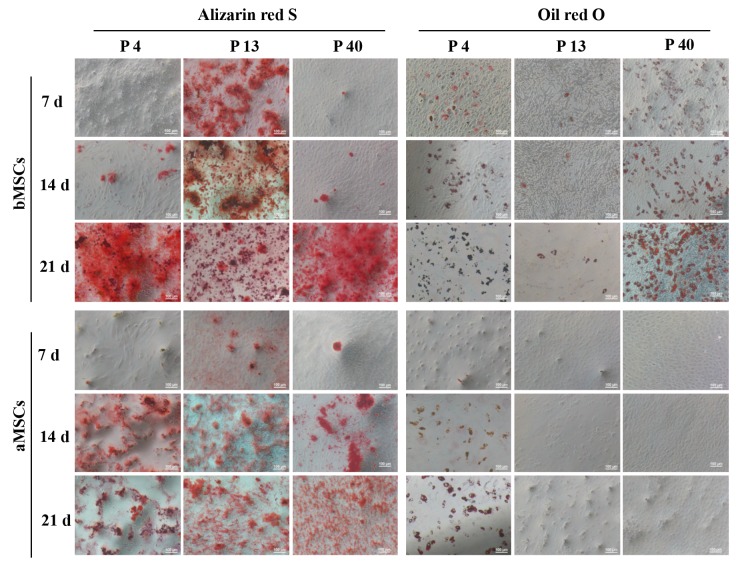
Differentiation of bMSCs and aMSCs. At different passages, bMSCs and aMSCs were cultured in osteogenic and adipogenic induction media for 21 days and stained with Alizarin Red S and Oil Red O on day (d) 7, 14, and 21, respectively.

The differentiation potential of bMSCs and aMSCs following long-term subculture was characterized regarding the osteogenic and adipogenic differentiation. As shown in Figure 5, after the osteogenic and adipogenic induction, formations of calcium-rich mineralized nodules and intracellular lipid vacuoles were detected by Alizarin Red S and Oil Red O staining, respectively. It was found that bMSCs at passage 13 were much more efficient in depositing the mineralized matrix on days 7 and 14 than those at passages 4 and 40. After 21 days of induction, bMSCs at all passages showed very strong Alizarin Red S staining. In contrast, bMSCs were prone to undergo adipogenic differentiation into mature adipocytes at passages 4 and 40, but not at passage 13. Interestingly, the efficiency of calcium deposition for aMSCs showed no apparent difference at different passages. However, while aMSCs displayed positive staining of Oil Red O at passage 4, lipid vacuoles could not be discerned at passages 13 and 40 within 21 days of induction. Together, bMSCs maintained their differentiation potential after long-term subculture and aMSCs lost the adipogenic differentiation upon subculture.

### 3.5. Immunophenotype of bMSCs and aMSCs

**Figure 6:**
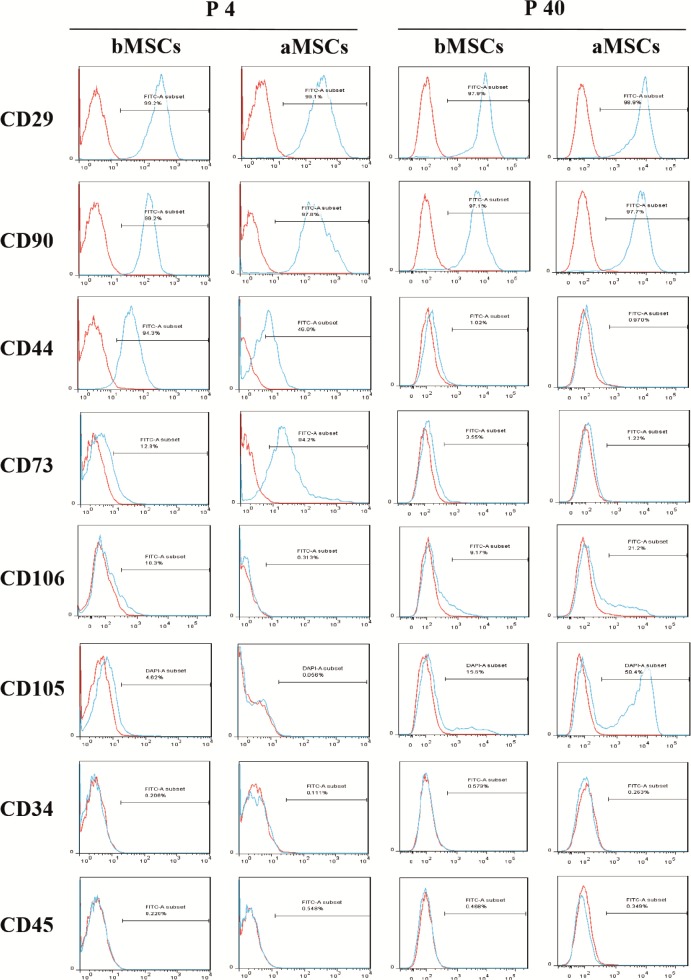
Analysis of surface makers of bMSCs and aMSCs. At passages 4 and 40, bMSCs and aMSCs were cultured for 5 days and then subjected to flow cytometric analysis to detect the expression of various surface markers.

To further characterize MSCs, cell surface marker proteins of bMSCs and aMSCs were examined by flow cytometric analysis. As shown in Figure 6, both bMSCs and aMSCs at passage 4 had no expression of hematopoietic progenitor cell marker CD34, panleukocyte marker CD45, or endoglin marker CD105, and more than 97% of both cell types expressed typical MSCs marker proteins including CD29 (integrin 1 chain) and CD90 (yTh-1). While CD44 and CD73 were present in both bMSCs and aMSCs, the percentage of CD44+ bMSCs was significantly larger than that of CD44+ aMSCs (94.3% vs. 46.0%) and the percentage of CD73+ cells was much greater for aMSCs than bMSCs (94.2% vs. 12.9%). Remarkably, 10.3% of bMSCs were found to express CD106 (vascular cell adhesion molecule-1, VCAM-1), which was not detected in aMSCs. At passage 40, the expression of CD29, CD90, CD34, and CD45 for both bMSCs and aMSCs was not distinct from that at passage 4. However, almost no expression of CD44 and CD73 and positive expression of CD105 (15.6% in bMSCs and 58.5% in aMSCs) was found at passage 40. Moreover, 21.2% of aMSCs became positive for CD106, while no significant difference was seen for bMSCs between passages 4 and 40. Collectively, long-term subculture could effect the immunophenotype of both bMSCs and aMSCs.

## 4. Discussion

In the present study, bMSCs and aMSCs were isolated from rats and then subjected to adherent subculturing. These cells were characterized in parallel to confirm their identity as authentic MSCs concerning cell morphology, proliferation, multilineage differentiation potential, colony-forming capability, and immunophenotyping during a long-term culture of 220 days. It was found that both bMSCs and aMSCs demonstrated similar typical bifroblast-like morphology in primary culture and the long-term culture made both types of cells smaller. Importantly, both cell types had great proliferative potential and grew faster following the continuous passaging. The colony formation ability of both types of MSCs and the differentiation potential of aMSCs decreased following subculture. In general, bMSCs showed greater potential in cell proliferation, colony formation, and differentiation. Moreover, the distinct expression of CD44, CD73, and CD106 was noticed between bMSCs and aMSCs and the long-term subculturing affected the expression of several surface markers, especially CD105, CD44, and CD73. This comparative study offers new insights into the differences between bMSCs and aMSCs, which have not been reported before.

MSCs-derived bone marrow and adipose tissue are most widely studied and applied in clinical settings. Current protocols for isolating aMSCs are mainly based on enzymatic digestion of adipose tissue samples, followed by adherent culture (Estes et al., 2010; Sugii et al., 2011) . For bMSCs, there are two most frequently applied methods, namely direct adherent culture based on differential adhesive properties of different cell types in bone marrow cell preparation (Huang et al., 2015) and density gradient purification followed by adherent culture (Sung et al., 2008) . However, Bortolotti et al. (2015) reported that bMSCs isolated by the former method tended to preserve better cell viability and paracrine secretion. For a straightforward comparison between bMSCs and aMSCs, cells were derived from bone marrow and adipose tissue preparations via direct adherent culture in the present study.

MSCs normally display fibroblast-like spindle shape in culture. However, the rat MSCs isolated in the present study were shorter and wider than those from humans (Sugii et al., 2011) , rabbits (Lee et al., 2014) , and goats (Mohamadfauzi et al., 2015) , but consistent with rat or mouse MSCs as reported by others (Peng et al., 1995; Huang et al., 2015) . Moreover, bMSCs and aMSCs showed similar shapes in the present study and both slightly transformed with culture time. Hence, the morphology of MSCs varies among species, but not tissue origins.

The self-renewal potential of MSCs was evaluated via long-term subculturing for 220 days. First, cell proliferation was characterized for both MSCs and compared. The proliferation rate of bMSCs and aMSCs increased first, then decreased, and finally stabilized following subculture during the whole culture period. This may have been caused by the increasing level of SA β-gal following the long-term subculture (Li et al., 2012; Gu et al., 2016) or the changing of passage-specific proteins (Çelebi et al., 2009). Nonetheless, bMSCs had a stronger capability to proliferate within early (<40 days) and late stages (>120 days) of subculturing than aMSCs, which was not consistent with results for human bMSCs and aMSCs (Guneta et al., 2016) . This discrepancy may be due to different starting cell densities applied in different studies (Meirelles and Nardi, 2003) , or species-specific differences. Within a total of 220 days, both bMSCs and aMSCs achieved about 150 PD (>1 × 10^45^ cells), indicating the sustainable potential of self-renewal during ex vivo expansion. In applying MSCs in clinical settings, a quantity of 1 × 10^9^ cells is generally required, thus making both bMSCs and aMSCs promising in therapeutic applications. Second, colony-forming capability, an important index of self-renewal, was analyzed for bMSCs and aMSCs. It was found that both cell types showed declined colony forming capability following passaging before passage 13, and bMSCs generally had higher CFU-Fs as compared to aMSCs. Third, the lineage-specific differentiation was assessed. While bMSCs maintained the multipotent potential of differentiating into osteoblasts and adipocytes following long-term subculture, aMSCs displayed no adipogenic differentiation after 32 PD. Meirelles and Nardi (2003) reported that murine bMSCs could sustain self renewal and multipotent potential for over 50 passages (equivalent to ~150 PD, over 8 months). Muraglia et al. (2000) as well as Gu et al. (2016) demonstrated that human bMSCs lost the adipogenic differentiation capacity after extended subculturing (22 PD). However, the present study represents the first study to compare between bMSCs and aMSCs. The loss of the differentiating potential of aMSCs after long-term subculture represents a limiting factor in using these cells in therapeutic applications. Some unspecified proteins may be responsible for the loss of differentiation potential of aMSCs (Çelebi et al., 2009). Supplementation of factors such as FGF-2 (Bianchi et al., 2003) , TGF-β, and PGE2 (Wu et al., 2016) during expansion culture of MSCs may be an effective way to ameliorate this.

Further, the immunophenotype of bMSCs and aMSCs was study by flow cytometric analysis of surface molecular markers during the long-term culturing. Overall, both bMSCs and aMSCs ubiquitously expressed CD29 and CD90 and did not express CD34 and CD45 independent of passages. This is consistent with the reported cell surface marker profile of rat bMSCs (Ran et al., 2009) , as well as the definition of human MSCs by the International Society for Cellular Therapy (ISCT) (Dominici et al., 2009) . However, neither bMSCs nor aMSCs expressed CD105 at passage 4, which is apparently inconsistent with some reports (Marappagounder et al., 2012; Otte et al., 2013) as well as the definition of human MSCs by the ISCT. However, the expression of CD105 in rabbit MSCs was found undetectable in one study (Lee et al., 2014) . Moreover, low to no expression of CD105 was also reported in human MSCs (Li et al., 2012; Tancharoen et al., 2017) . Hence, the expression of surface markers can in fact be dependent on several factors such as the species as well as the process protocols. Intriguingly, following subculture, bMSCs and aMSCs showed a fairly noticeable declination in the expression of CD44 and CD73, but upregulation of CD105. Only late-passage aMSCs showed an increased expression of CD106. These changes in the immunophenotype of MSCs may effect their functions (Bara et al., 2014) . For instance, CD105- MSCs exhibited enhanced adipogenic potential (Jiang, et al. 2010; Anderson, et al., 2013) ; it has been suggested that the expression of CD106 on MSCs might be linked to the lineage-specific differentiation potential of MSCs (Fukiage et al., 2008; Chen et al., 2015) .

In the present study, MSCs were isolated from rat bone marrow and adipose tissue and subjected to a long-term subculture for 220 days. Within 220 days, both bMSCs and aMSCs demonstrated varying proliferation rate, colony-forming capability, multilineage differentiation potential, and immunophenotype with the subculturing. While both types of MSCs proliferated faster following the long-term subculture, bMSCs showed higher proliferation rate, colony-forming capability, and multilineage potential compared to aMSCs. These results highlight the profound differences in characteristics and functions of MSCs derived from different tissue origins.

## Acknowledgments

This work was supported by the National Natural Science Foundation of China (81671841), the National Special Fund for the State Key Laboratory of Bioreactor Engineering (2060204), and the Natural Science Foundation of Shanghai (16ZR1408700).
